# Global, regional, and national burden of osteoarthritis among middle-aged and older adults: estimates from the global burden of disease study 2021 and projections to 2050

**DOI:** 10.3389/fmed.2025.1696929

**Published:** 2025-12-16

**Authors:** Yichen Wang, Xing Tang, Jie-Ru Peng, Yuanhong Deng, Shoujen Lan, Yeayin Yen, Yong Tang

**Affiliations:** 1 School of Health and Rehabilitation, Chengdu University of Traditional Chinese Medicine, Chengdu, China; 2 College of Acupuncture-Moxibustion and Tuina, Chengdu University of Traditional Chinese Medicine, Chengdu, China; 3 Department of Epidemiology and Biostatistics, West China School of Public Health and West China Fourth Hospital, Sichuan University, Chengdu, China; 4 Department of Healthcare Administration, Asia University, Taichung, China

**Keywords:** osteoarthritis, middle-aged and older adults, global burden of disease (GBD), temporal trends, projection

## Abstract

**Background:**

Osteoarthritis (OA) is a leading cause of pain, disability, and reduced quality of life among middle-aged and older adults. Understanding its global epidemiological patterns and attributable risk factors is essential for public health planning. This study quantified the burden of OA among adults aged 55 years and older, evaluated temporal trends from 1990 to 2021, and projected future trends through 2050.

**Methods:**

Data on OA incidence, prevalence, and disability-adjusted life years (DALYs) were obtained from the Global Burden of Disease Study 2021 (GBD 2021). OA cases were defined by symptomatic and radiographically confirmed criteria. Age-standardized rates (ASRs) per 100,000 population were calculated, and temporal trends were analyzed using estimated annual percentage change (EAPC). High body mass index (BMI ≥25 kg/m^2^) was assessed as an attributable risk factor. Age–period–cohort (APC) models were applied to assess independent effects of age, calendar period, and birth cohort. Bayesian APC (BAPC) models projected OA burden through 2050. Analyses were stratified by sex, age group, region, and Sociodemographic Index (SDI) quintile.

**Results:**

In 2021, 23.86 million older adults were newly diagnosed with OA globally (ASIR 1,610.95 per 100,000), with 453.56 million living with OA (ASPR 30,395.11 per 100,000) and 16.05 million DALYs (ASDR 1,075.70 per 100,000) among adults aged 55 years and older. All metrics showed significant upward trends since 1990. High-SDI regions exhibited the highest burden. Age, period, and cohort analyses revealed rising incidence peaked at 55–59 years, simultaneously, OA risks in more recent birth cohorts and persistent increases with advancing age. Females consistently experienced higher rates than males. Globally, 35.8% of OA-related DALYs were attributable to high BMI, with the greatest burden in High-income North America. Projections indicate further increases by 2050 (ASIR 9%, ASPR 14%, ASDR 12%).

**Conclusion:**

OA imposes a substantial and increasing health burden on middle-aged and older adults globally, with notable variations across regions, sex, age, and SDI levels. High BMI contributes significantly to this burden. Our projections indicate continued growth in OA incidence and disability, emphasizing the need for targeted prevention and intervention strategies to reduce risk factors and improve population health outcomes.

## Introduction

Osteoarthritis (OA) is a chronic, degenerative joint disorder that predominantly involves the cartilage of weight-bearing joints, with the knee being most frequently affected, followed by the hip, hands, spine, and other anatomical sites (1). Clinically, OA is characterized by joint pain, stiffness, and restricted mobility, all of which substantially impair physical function and diminish the life quality of patients (2). Driven by global trends in population aging and rising obesity rates, the prevalence of osteoarthritis continues to increase. In 2020, OA affected an estimated 7.6% of the global population, with more than 595 million individuals living with OA, and if this trend persists, the number of OA cases is projected to rise by 60%−100% by 2050 (3). Moreover, OA substantially contributes to disability and is expected to become the leading cause of disability worldwide by 2030 (4).

In addition to its health impact, OA imposes a substantial economic burden on both individuals and healthcare systems, encompassing both direct medical costs and indirect costs such as productivity losses and caregiving time (5). At the patient level, knee OA contributes to a considerable incremental healthcare burden over the 5 years following diagnosis, with each patient incurring approximately 16.8 additional healthcare consultations, 0.7 inpatient days, 420 defined daily doses of prescribed medications, and 21.8 net disability days (6). These increased healthcare needs translate into substantial financial implications. Globally, the average annual cost per individual with OA has been estimated to range from $700 to $15,600 (2019 USD), depending on healthcare system structure and disease severity. In countries with established market economies, the total cost of OA is estimated to account for approximately 1%−2.5% of the gross national product (4). Moreover, projections from a recent national-level study in Australia underscore the growing financial burden of OA in the coming decades. Based on current average annual healthcare expenditures of AU$2,100 per person with OA, total annual health system spending on these conditions is conservatively forecast to exceed AU$11.92 billion by 2040 in Australia (7).

The etiology and pathogenesis of OA are complex and multifactorial. Among various contributing factors, aging is recognized as one of the most critical. Age-related biological changes, including chondrocyte senescence, the accumulation of pro-inflammatory cytokines such as interleukin-1*β* (IL-1*β*), tumor necrosis factor (TNF), and interleukin-6 (IL-6) (8), as well as metabolic disturbances such as abnormal lipid metabolism (9), directly accelerate the progression of OA. These alterations contribute to chronic inflammation in joint tissues, including cartilage, synovium, and subchondral bone, and impair their repair capacity (10). Around 35% of the global older population experiences symptomatic (painful, disabling) OA (11). Furthermore, advancing age is significantly associated with increased disease severity, with older adults showing more pronounced abnormalities in structural parameters of the knee joint, including the sulcus angle and tibial tubercle to trochlear groove distance (12). Notably, recent epidemiological studies have indicated a temporal shift in the age-specific prevalence pattern of OA, with the peak prevalence also occurring between 55 and 59 years of age (13). These findings underscore the importance of focusing on the epidemiological characteristics, disease burden, and trends of OA among older populations, particularly those aged 55 years and above, which is crucial for developing targeted prevention and management strategies.

The Global Burden of Disease (GBD) study has become an essential resource for comprehensively understanding the epidemiological patterns and health impacts of a wide range of diseases and injuries. The most recent GBD 2021 update systematically evaluates the burden of 371 diseases and injuries using standardized metrics such as incidence, prevalence, disability-adjusted life years (DALYs), and years lived with disability (YLDs) (14, 15). These data support an in-depth examination of the distribution and temporal trends of disease burden across diverse populations, geographic regions, and periods. Leveraging the comprehensive GBD 2021 dataset, this study aims to quantify the global, regional, and national burden of OA among individuals aged 55 years and older, a population increasingly affected by age-related musculoskeletal conditions. Simultaneously, our study investigates temporal trends and demographic variations stratified by sex, age group, GBD region, country or territory, and Sociodemographic Index (SDI), and projects the epidemiological profile and disease burden of OA through the year 2050.

## Methods

### Data sources

All data analyzed in this study were obtained from the GBD 2021, accessible via the Global Health Data Exchange (GHDx) online platform (http://ghdx.healthdata.org/gbd-results-tool), publicly released by the Institute for Health Metrics and Evaluation (IHME) at the University of Washington. GBD 2021 provides the most up-to-date epidemiological estimates of data on a total of 371 diseases and 88 risk factors across 204 countries and territories from 1990 to 2021. The database includes standardized data on disease incidence, prevalence, mortality, and DALYs, stratified by age, sex, year, and geographical location. For this study, we extracted annual estimates related to OA, including incidence, prevalence, and DALYs, disaggregated by sex, 5-year age groups (from age 55 and above), and by region and SDI quintiles. The data were generated through systematic reviews, survey and registry data, and Bayesian meta-regression modeling (DisMod-MR 2.1), ensuring internal consistency and comparability across locations and years. Detailed methodological documentation of GBD estimation procedures is available from IHME (14).

### Case definition

In the GBD 2021 framework, OA cases were defined as individuals with symptomatic OA confirmed by radiographic evidence, corresponding to Kellgren–Lawrence grades 2–4. This definition captures clinically relevant disease with established joint structural changes. The primary data sources for estimating the burden of hip and knee OA included cross-sectional, population-based health surveys from multiple countries, as well as administrative claims data, particularly from state-level health insurance databases in the United States. These datasets identified OA cases using the International Classification of Diseases, 10th Revision (ICD-10), with codes M16 for hip OA and M17 for knee OA. Beginning with GBD 2019, the case definition was expanded to include two additional anatomical sites: hand OA (ICD-10 code M18) and other joints OA (M19). These classifications were retained in GBD 2021 to allow for more comprehensive anatomical coverage and consistency across estimation cycles. All OA estimates were modeled using standardized case definitions to ensure cross-national comparability and temporal consistency (16).

### Risk estimation for high body mass index

High body mass index (BMI), defined as ≥25 kg/m^2^ for adults, was the only risk factor documented in GBD 2021with sufficient evidence to quantify its attributable burden for OA. The attributable DALYs were estimated using the comparative risk assessment framework. This incorporated exposure distributions, theoretical minimum risk exposure levels (TMRELs), and relative risks derived from epidemiological studies.

### Statistical analysis

Our study described OA burden metrics (incidence, prevalence, and DALYs) by age, sex, year, region, and SDI quintile. Temporal trends were evaluated using estimated annual percentage change (EAPC), calculated by fitting a linear regression model to the natural logarithm of the age-standardized rates (ASRs). To disentangle the temporal patterns of OA burden, we applied an age–period–cohort (APC) model to assess the independent effects of age, calendar period, and birth cohort. Additionally, a Bayesian age–period–cohort (BAPC) model was used to project future trends in OA burden among middle-aged and older adults. All statistical analyses and data visualizations were performed using R software (version 4.4.2).

The ASRs of per 100,000 population was calculated using the following formula:


$$ASR=\frac{\sum_{i=1}^{A}a_{i}w_{i}}{\sum_{i=1}^{A}w_{i}}\;\times \;100,000\;$$ (1)

Where *a*_i_ is the age-specific rate for the *i* th age group, w_*i*_ is the number of individuals in the standard population for the same age group, and A is the total number of age groups.

EAPC was calculated based on a regression model that describes the natural logarithm of the ASR over a specified period. The model takes the form Y = *α + β*X *+ ε*, where Y is the natural log of the ASR, X is the calendar year, α is the intercept, *β* is the slope (indicating the trend), and ε is the error term. The EAPC is computed using the formula:


$$\begin{array}{l} EAPC\;=\;100\;\times \;\left[\exp\left(\beta \right)-1\right] \end{array}$$ (2)

A 95% confidence interval (CI) for the EAPC was calculated using a linear regression model. An EAPC with a 95% CI entirely above or below zero was considered statistically significant.

APC models were applied to disentangle the temporal trends in OA burden into independent effects of age, calendar period, and birth cohort. The analysis was conducted using the intrinsic estimator (IE) method, which provides robust estimates while addressing the identification problem inherent to APC modeling. Relative risks (RRs) and 95% CIs were derived for each period and cohort relative to the reference groups.

To forecast future OA burden through 2050, BAPC modeling framework based on integrated nested Laplace approximation (INLA) we employed as implemented in the GBD forecasting approach. This method accounts for historical age-specific rates, period effects, and population projections from the UN World Population Prospects 2022. Projections were expressed as absolute counts and age-standardized rates for incidence (ASIR), prevalence (ASPR), and DALYs (ASDR).

## Results

### Global burden of osteoarthritis among older adults

As presented in Table 1, approximately 23.86 million (95% UI: 20.41–27.69 million) older individuals were newly diagnosed with OA worldwide in 2021, corresponding to an ASIR of 1,610.95 (95% UI: 1,232.32–2,036.39) per 100,000 population. From 1990 to 2021, the ASIR showed a statistically significant increasing trend, with an EAPC of 0.22 (95% CI: 0.19–0.24). Globally, 453.56 million (95% UI: 400.66–505.18 million) older individuals were living with OA in 2021, with an ASPR of 30,395.11 (95% UI: 26,587.13–34,279.51) per 100,000 population. The EAPC prevalence from 1990 to 2021 was 0.30 (95% CI: 0.27–0.32). The estimated number of DALYs associated with OA increased to 16.05 million (95% UI: 7.77–32.49 million) among the global older population in 2021. The corresponding ASDR was 1,075.70 (95% UI: 522.55–2,168.70) per 100,000 population, with an EAPC of 0.33 (95% CI: 0.30–0.36) since 1990.

**Table 1:** Global and SDI-specific trends in the number and age-standardized rates of incidence, prevalence, and DALYs for osteoarthritis among middle-aged and older adults, 1990–2021.

**Metric**	**Region/SDI Level**	**1990**		**2021**	
		**Number (N, 95% UI)**	**ASR (per 100 000, 95% UI)**	**Number (N, 9 5% UI)**	**ASR (per 100 000, 95% UI)**
Incidence	Global	10,099,033.47 (8,568,098.71–11,803,212.08)	1,492.97 (1,145.34–1,892.37)	23,858,140.28 (20,407,940.15–27,685,712.00)	1,610.95 (1,232.32–2,036.39)
	Low SDI	491,600.30 (411,439.94–579,500.76)	1,296.94 (992.27–1,652.18)	1,170,312.39 (984,850.25–1,373,856.56)	1,406.13 (1,078.22–1,779.47)
	Low-middle SDl	1,336,620.73 (1,122,276.52–1,580,214.11)	1,310.43 (998.46–1,666.66)	3,572,228.18 (3,027,659.28–4,199,071.13)	1,468.72 (1,119.10–1,862.22)
	Middle SDI	2,405,421.12 (2,021,898.90–2,830,567.67)	1,367.50 (1,036.91–1,742.69)	7,320,269.95 (6,207,075.79–8,560,702.60)	1,547.35 (1,174.61–1,969.86)
	High-middle SDl	2,588,392.39 (2,201,908.84–3,033,903.12)	1,490.16 (1,141.92–1,883.95)	5,513,417.00 (4,725,326.68–6,395,384.08)	1,599.55 (1,222.10–2,018.39)
	High SDI	3,265,545.30 (2,810,941.13–3,808,216.04)	1,793.40 (1,387.71–2,258.20)	6,261,522.65 (5,422,979.25–7,213,885.60)	1,902.45 (1,469.08–2,392.32)
Prevalence	Global	185,881,360.20 (164,341,334.80–207,273,096.40)	28,032.71 (24,554.46–31,637.22)	453,562,905.70 (400,659,708.50–505,177,611.60)	30,395.11 (26,587.13–34,279.51)
	Low SDI	7,954,402.26 (6,977,829.13–8,953,140.06)	22,278.38 (19,464.65–25,363.06)	19,493,483.04 (17,158,914.48–21,924,865.08)	24,570.89 (21,478.09–27,845.29)
	Low-middle SDl	22,418,385.71 (19,714,042.02–25,145,130.53)	23,094.31 (20,105.80–26,268.72)	62,602,519.51 (55,221,670.97–70,126,508.80)	26,457.69 (23,072.95–29,999.65)
	Middle SDI	43,856,989.84 (38,510,327.84–49,202,253.78)	26,062.96 (22,740.74–29,588.02)	137,829,464.60 (121,244,280.10–153,976,106.70)	29,696.83 (25,927.08–33,621.13)
	High-middle SDl	48,846,603.56 (43,052,184.86–54,467,719.40)	28,634.70 (24,984.91–32,315.10)	107,786,539.70 (94,941,200.18–120,119,233.50)	30,835.04 (26,878.76–34,788.77)
	High SDI	62,594,982.09 (55,878,782.65–69,379,598.37)	32,660.86 (28,696.43–36,747.13)	125,456,942.30 (112,257,322.50–138,537,801.10)	34,760.89 (30,578.07–39,040.09)
DALYs	Global	6,525,963.43 (3,164,698.27–13,190,234.34)	984.19 (479.05–1,986.22)	16,050,199.63 (7,768,330.37–32,489,948.10)	1,075.70 (522.55–2,168.70)
	Low SDI	268,766.92 (129,709.37–538,313.91)	751.03 (364.57–1,508.46)	667,264.44 (322,565.49–1,335,115.13)	840.12 (407.96–1,683.79)
	Low-middle SDl	761,160.08 (366,724.98–1,530,025.12)	782.95 (379.95–1,575.99)	2,157,784.70 (1,042,570.72–4,351,949.44)	911.43 (442.40–1,833.35)
	Middle SDI	1,512,831.51 (728,023.31–3,042,347.34)	898.11 (434.61–1,808.20)	4,830,878.26 (2,333,153.62–9,755,060.18)	1,040.75 (504.01–2,094.16)
	High-middle SDl	1,718,100.97 (833,361.25–3,474,587.60)	1,007.14 (489.62–2,037.71)	3,823,673.06 (1,837,321.96–7,768,966.26)	1,093.85 (529.26–2,211.30)
	High SDI	2,257,728.89 (1,103,269.67 −4,571,301.17)	1,177.26 (575.43–2,368.27)	4,556,625.33 (2,225,901.71–9,247,304.25)	1,261.97 (615.31–2,543.18)

ASR, age-standardized rate; SDI, Socio-demographic index; UI, uncertainty interval.

### Regional and national burden of osteoarthritis among older adults

These global patterns showed substantial variation across different SDI regions. High-SDI regions exhibited the highest ASIR, ASPR and ASDR, with values of 1,902.45 (95% UI: 1,469.08–2,392.32), 34,760.89 (95% UI: 30,578.07–39,040.09), and 1,261.97 (95% UI: 615.31–2,543.18) per 100,000 population, respectively. In contrast, low-SDI regions had the corresponding rates, at 1,406.13 (95% UI: 1,078.22–1,779.47), 24,570.89 (95% UI: 21,478.09–27,845.29), and 840.12 per 100,000 population (95% UI: 407.96–1,683.79), respectively.

**Figure 1:**
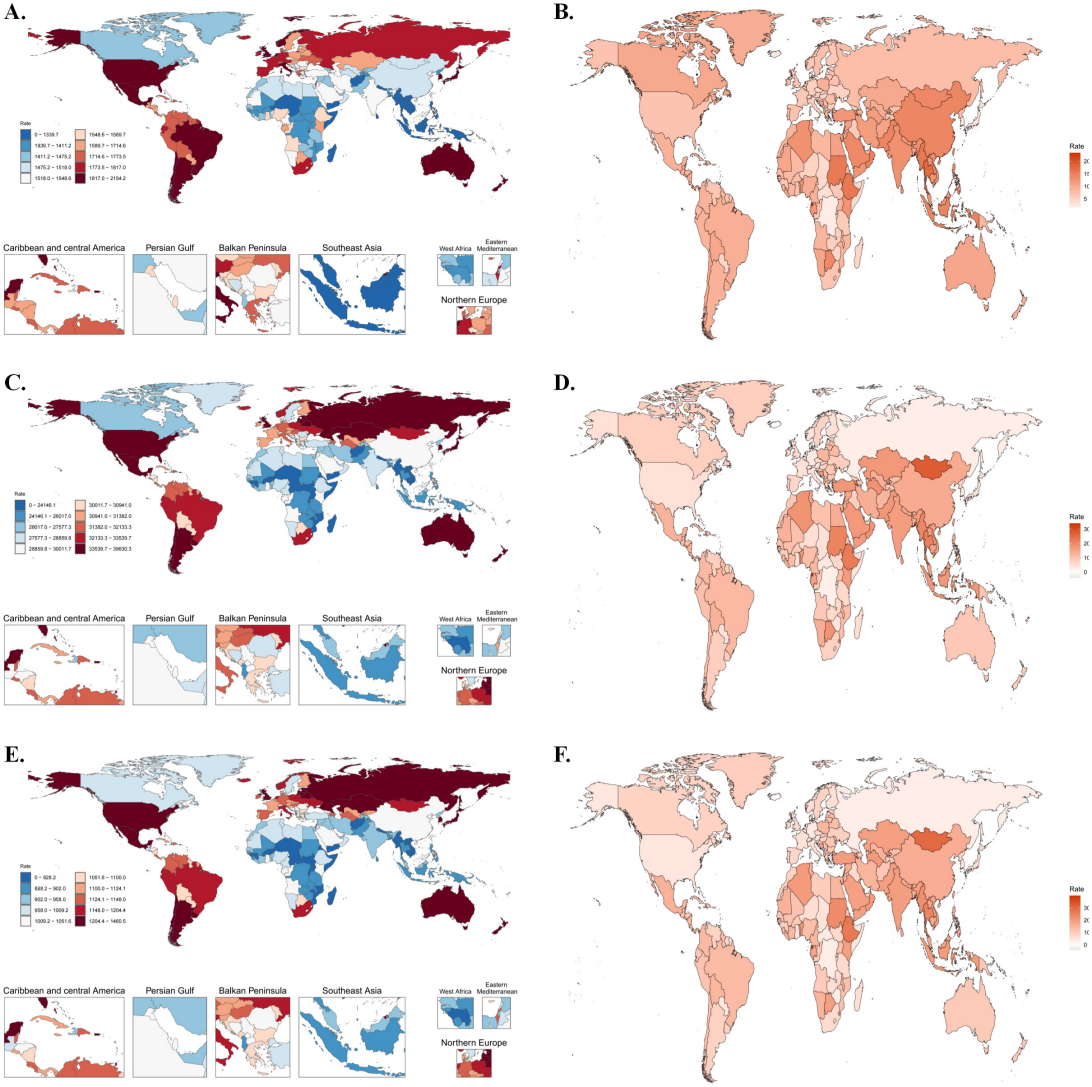
Global disease burden and changes in older osteoarthritis in 204 countries and regions. **(A)** Age-standardized incidence rate (ASIR) of osteoarthritis in the older adult, 2021. **(B)** Percentage change in age-standardized incidence rate (ASIR) of older osteoarthritis, 1990–2021. **(C)** Age-standardized prevalence rate (ASPR) of osteoarthritis in the older population, 2021. **(D)** Percentage change in age-standardized incidence rate (ASIR) of older osteoarthritis, 1990–2021. **(E)** Age-standardized disability-adjusted life years (DALYs) rate of osteoarthritis in the older population, 2021. **(F)** Percentage change in age-standardized disability-adjusted life years (DALYs) rate of older osteoarthritis, 1990–2021.

As depicted in Figure 1, among the 21 GBD regions, the highest burden of OA among the older population was observed in the High-income Asia Pacific, High-income North America, and Australasia regions. In contrast, the lowest burden was recorded in South Asia. The highest ASIR and ASPR of OA in 2021 were predominantly reported in the Republic of Korea, Brunei Darussalam, Singapore, the United States, and Japan. Notably, the highest ASDR was observed in Sweden, Ethiopia, Lebanon, and Lesotho.

### Sex- and age-specific burden of osteoarthritis among older adults

**Figure 2:**
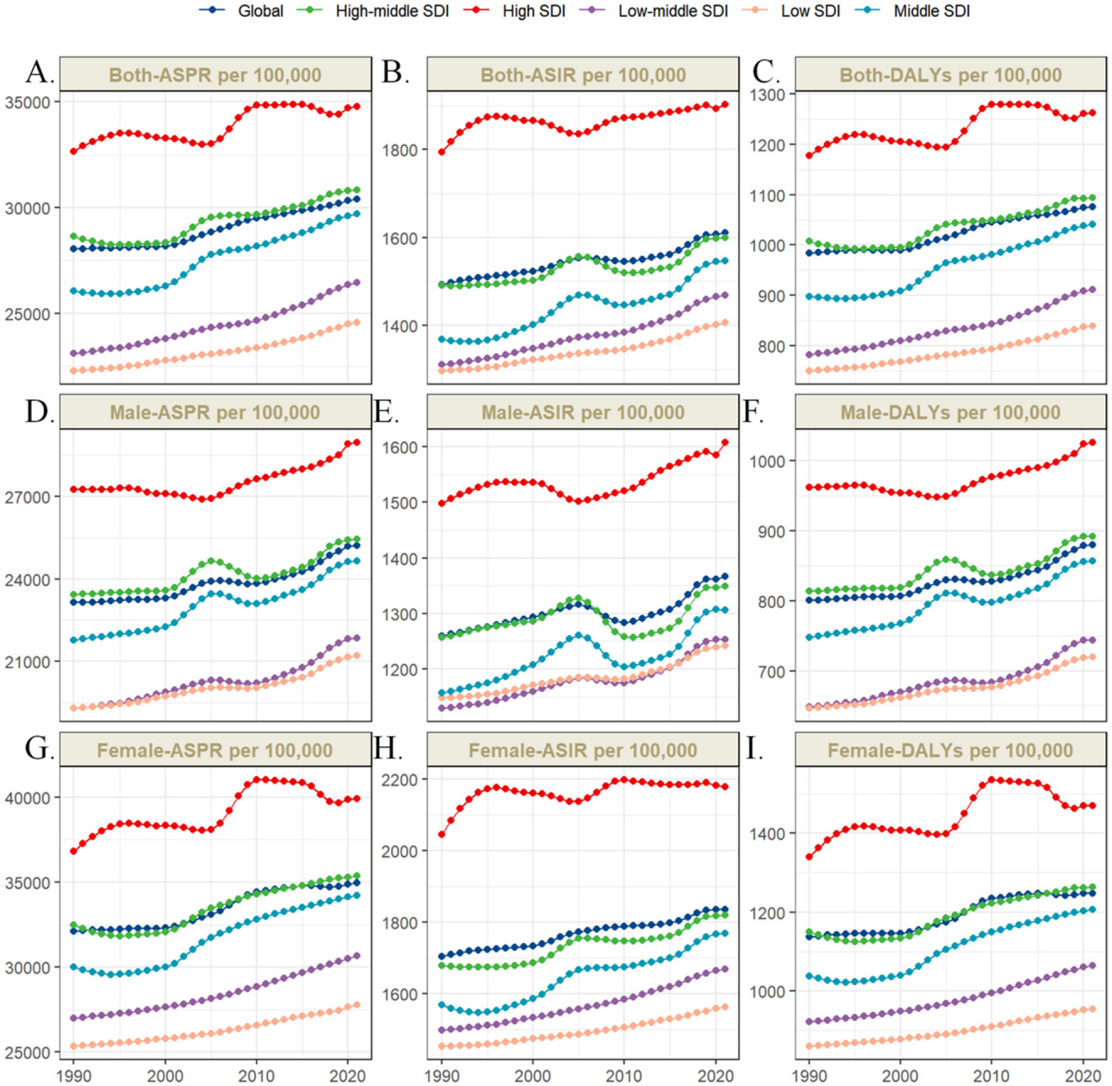
Trends in ASIR, ASPR and ASDR of osteoarthritis among older adults globally and across five sociodemographic index regions from 1990 to 2021. Between 1990 and 2021, the global age-standardized incidence **(B, E, H)**, prevalence **(A, D, G)**, and DALY **(C, F, I)** rates of osteoarthritis among older adults increased steadily. Similar upward trends were observed across all five SDI regions.

**Figure 3:**
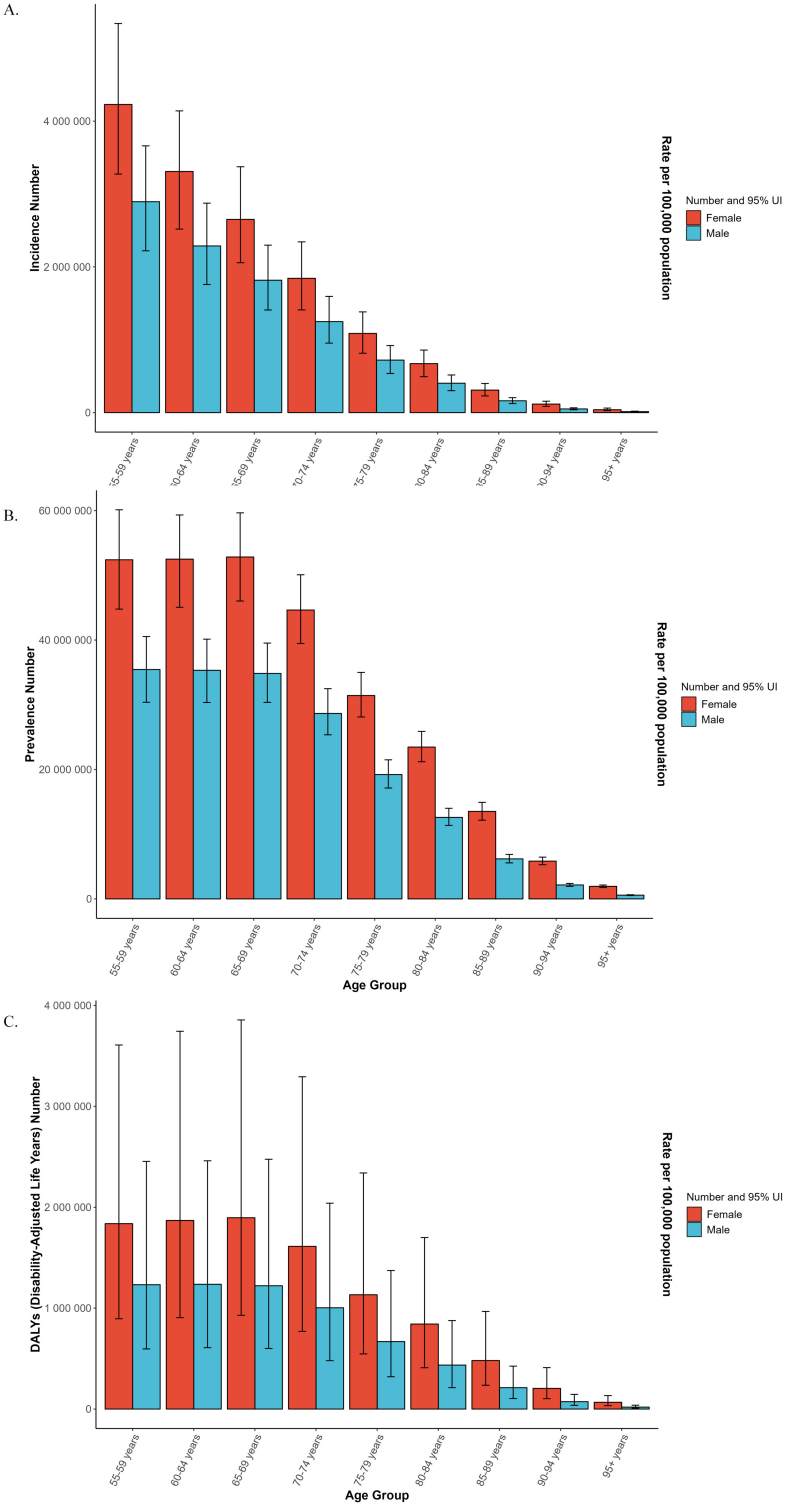
Global age-standardized prevalence **(B)**, incidence **(A)**, and DALY **(C)** rates of osteoarthritis among older adults in 2021, stratified by sex and age group.

As illustrated in Figure 2, between 1990 and 2021, the global ASIR, ASPR and ASDR of OA among individuals aged 55 years and older demonstrated a sustained upward trajectory in both sexes. Similar temporal patterns were observed across all SDI regions. As illustrated in Figure 3, across comparable time points and geographic regions, females consistently exhibit higher ASIR, ASPR and ASDR of OA than males.

**Figure 4:**
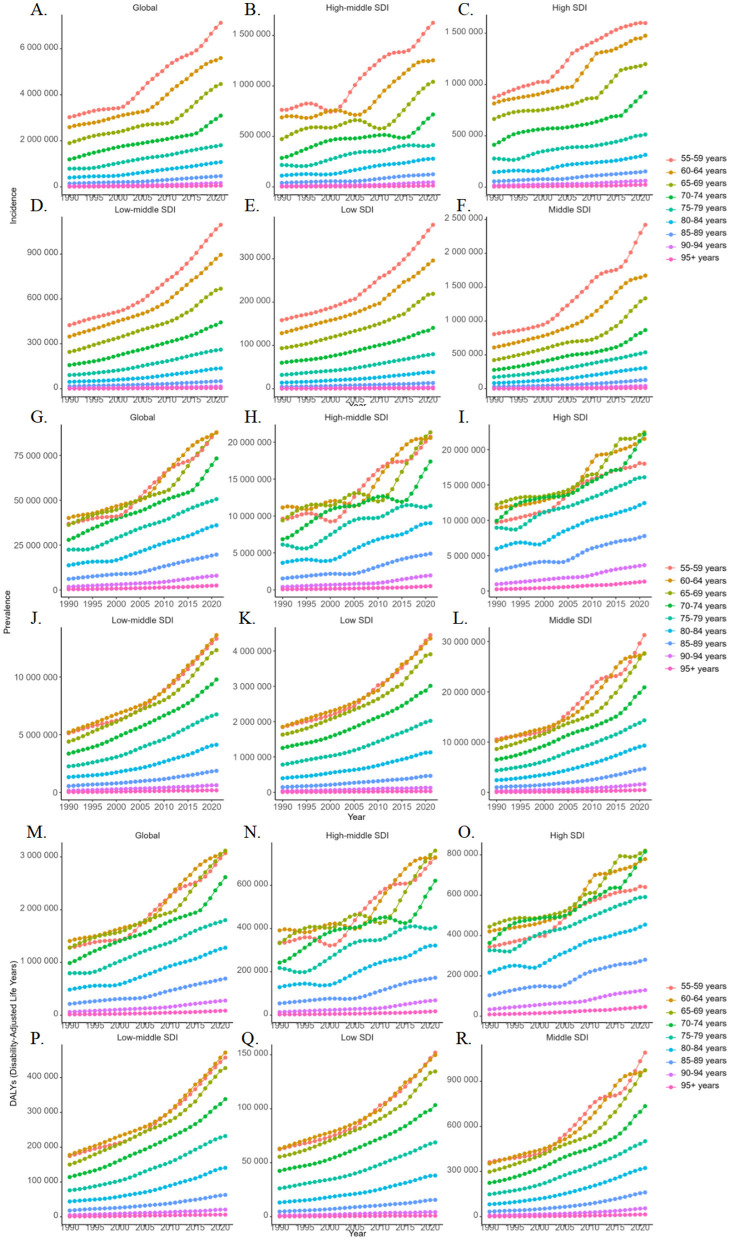
Age-specific group trends in the age-standardized prevalence **(G–L)**, incidence **(A–F)**, and DALY **(M–R)** rates of osteoarthritis among older adults globally and across five sociodemographic index regions from 1990 to 2021.

Regarding age-specific patterns in disease burden (Figure 4), although OA has traditionally been characterized as a degenerative condition predominantly affecting the older population, our findings reveal that the incidence rate was highest in the 55–59 age group, exceeding that of individuals typically classified as older population (aged 60 years and older). Across all SDI regions, the most notable increases in ASIR were observed in the 55–59 age group, followed by the 60–64 age group, with incidence rates in the 65–69 age group approaching comparable levels. Similarly, notable increases in ASPR and ASDR were observed in the 55–59, 60–64, and 65–69 age groups, with the most prominent rise in DALY rates for the 55–59 age group occurring in middle-SDI regions.

### Association between the burden of osteoarthritis and SDI

**Figure 5:**
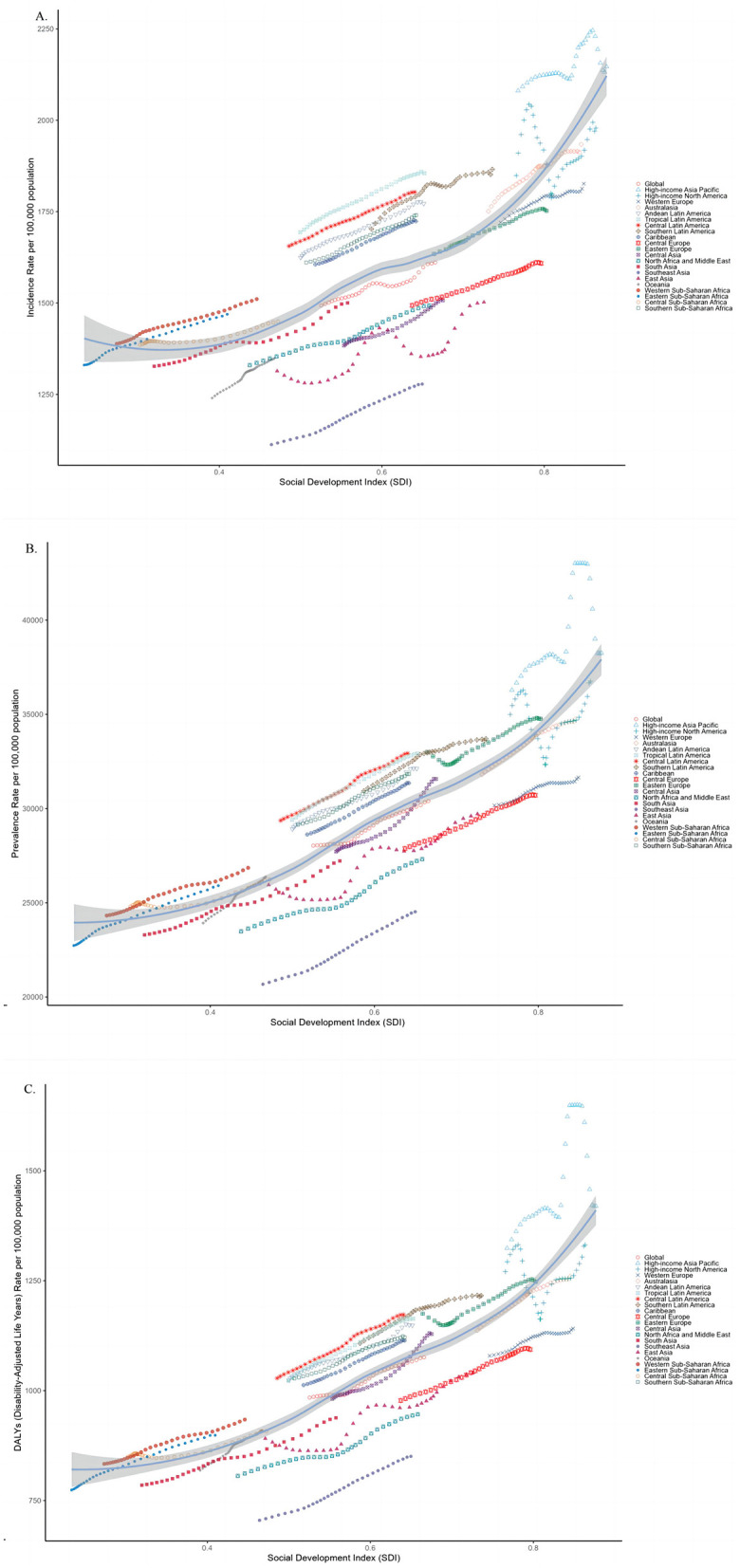
Relationship between the sociodemographic index and age-standardized **(B)**, incidence **(A)**, and DALY **(C)** rates of osteoarthritis among older adults across the 21 GBD regions.

According to Figure 5, between 1990 and 2021, at both the global and regional levels, a positive correlation was observed between SDI and the age-standardized rates of osteoarthritis among older adults. A strong positive correlation was found between SDI and ASPR (*r* = 0.828, *p* < 0.001), as well as between SDI and the ASDR (*r* = 0.840, *p* < 0.001). Additionally, a moderate but statistically significant positive correlation was noted between SDI and ASIR (*r* = 0.743, *p* < 0.001).

### High BMI-attributable burden of osteoarthritis

**Figure 6:**
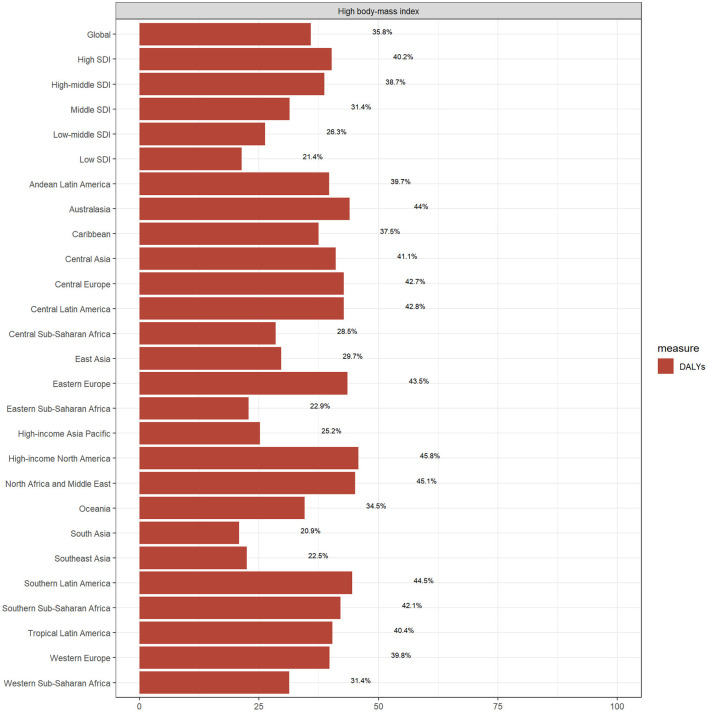
Proportion of DALYs attributable to high body mass index among older adults with osteoarthritis in 2021 across SDI and GBD regions.

High BMI is the only risk factor for OA documented within the GBD framework and it plays a critical role in the overall OA burden. As illustrated in Figure 6, in 2021, 35.8% of global OA-related DALYs were attributable to high BMI. This proportion varied across regions and sociodemographic levels. The highest burden was observed in High-income North America, where over 45.8% of OA-related DALYs were attributable to high BMI, more than twice the proportion seen in South Asia (20.9%), which had the lowest attributable burden.

### Projected trends in the burden of osteoarthritis among older Adults through 2050

**Figure 7:**
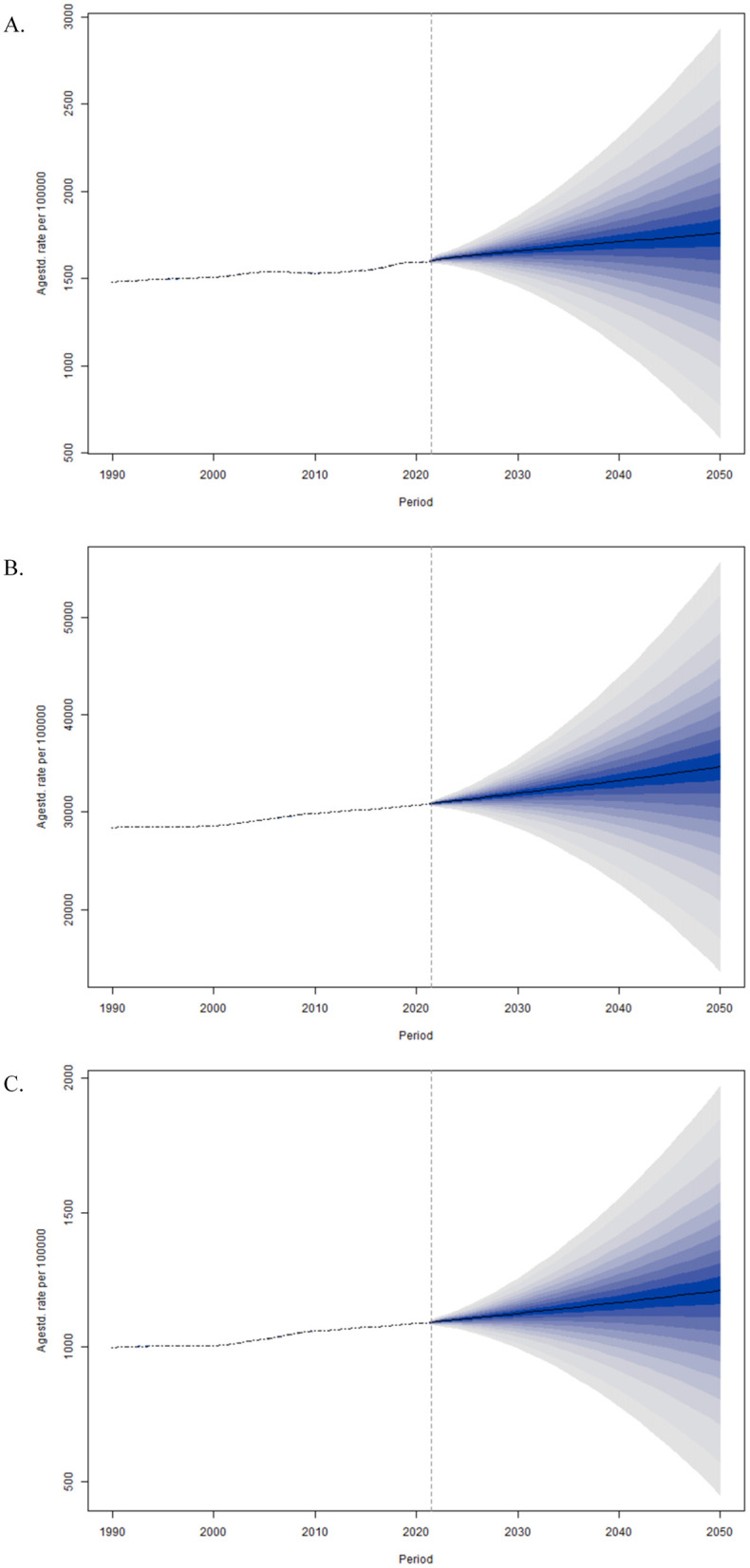
Temporal trends in age-standardized prevalence, incidence, and DALY rates of osteoarthritis among older adults globally from 1990 to 2050, projected using Bayesian age-period-cohort (BAPC) modeling. **(A)** Global age-standardized incidence rate of osteoarthritis in older adults, 1990–2050. **(B)** Global age-standardized prevalence rate of osteoarthritis in older adults, 1990–2050. **(C)** Global age-standardized DALY rate of osteoarthritis in older adults, 1990–2050.

By the BAPC model, we projected the global ASIR, ASPR and ASDR of OA among older adults over the next 30 years. As shown in Figure 7A, the global ASPR is expected to reach 34,652 per 100,000 people by 2050, which is a 14% increase from 2021. Similarly, the ASIR is expected to rise to approximately 1,758 per 100,000 people by 2050, reflecting a 9% increase from 2021 (Figure 7B). Concurrently, the ASDR is estimated to reach 1,209 per 100,000 people by 2050, indicating a 12% increase from 2021 (Figure 7C). These projections indicate an ongoing upward trend in OA's global burden among older adults.

### Age, period, and cohort effects on the burden of osteoarthritis among older adults

**Figure 8:**
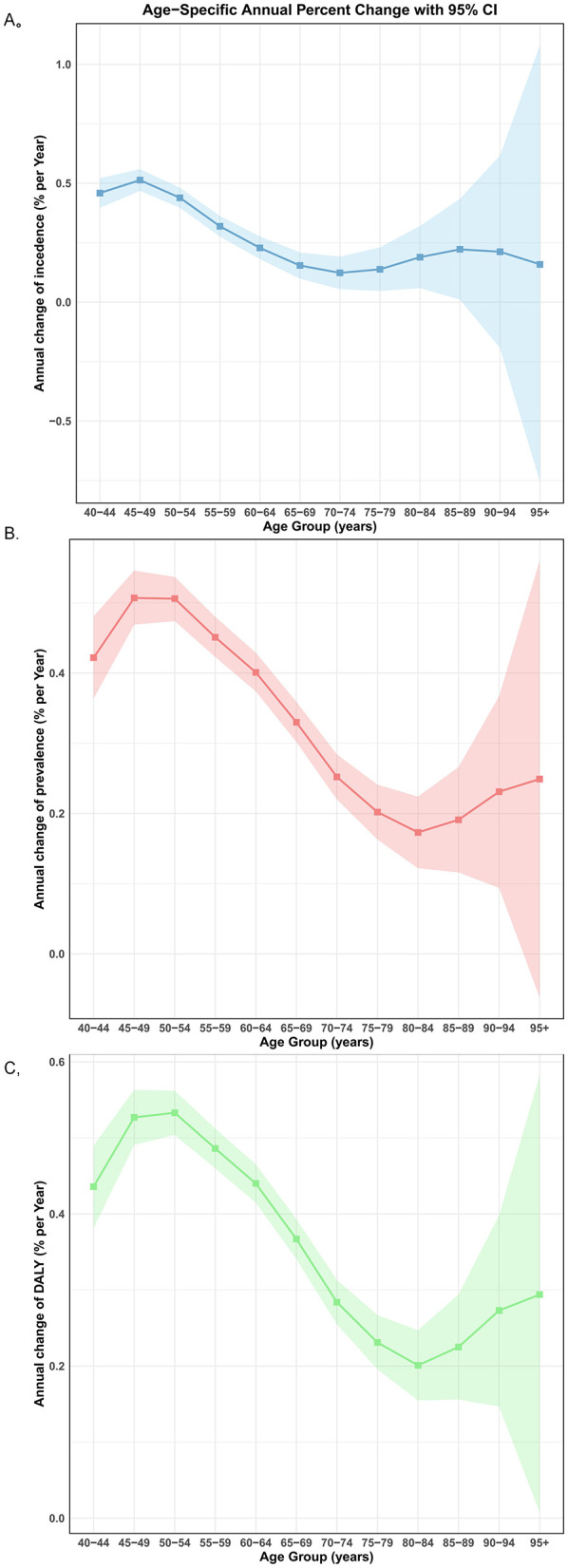
Local drifts of OA incidence, prevalence and DALY among older people during 1992–2021. **(A)** Local drifts of OA incidence (estimates from age-period-cohort models) for 9 age groups (55–60 to 95+ years), 1992 −2021. **(B)** Local drifts of OA prevalence (estimates from age-period-cohort models) for 9 age groups (55–60 to 95+ years), 1992 −2021. **(C)** Local drifts of OA DALY (estimates from age-period-cohort models) for 9 age groups (55–60 to 95+ years), 1992 −2021. The dots and shaded areas indicate the annual percentage change of OA incidence, prevalence and DALY (% per year) and the corresponding 95% CIs.

Figure 8 shows the annual percentage change in OA incidence, prevalence and DALY rates across age groups with the local drift estimated from the APC model, reflecting trends in birth cohort effects from ages 55–59 to ≥95 years. Globally, the incidence exhibited increasing trends across 55–59 to 85–89 age groups, while both prevalence and DALY rates increased significantly across all age groups (*p* < 0.001). For incidence, the greatest increase occurred in the 55–59 years group with an annual percentage change of 0.30 (95% CI 0.26–0.34), and the increasing trend attenuated with advancing age up to the 70–74 group. Notably, from ages 90–94 to ≥95 years, the percentage change was accompanied by substantially widened 95% confidence intervals encompassing zero, indicating that the observed changes were not statistically significant in the oldest age cohorts. For prevalence, the steepest increase was also observed in the 55–59 years group with annual percentage change of 0.43 (95% CI 0.41–0.46), the percentage change gradually decreased through to the 80–84 group (0.17, 95% CI 0.14, 0.21), followed by a slightly increase from 85–89 to ≥95 years. A similar pattern was observed for DALY, the percentage change declined progressively from 0.47 (95% CI: 0.44–0.49) in the 55–59 group to 0.20 (95% CI: 0.17–0.23) in the 80–84 group, then slightly increased from 85–89 to ≥95 years.

**Figure 9:**
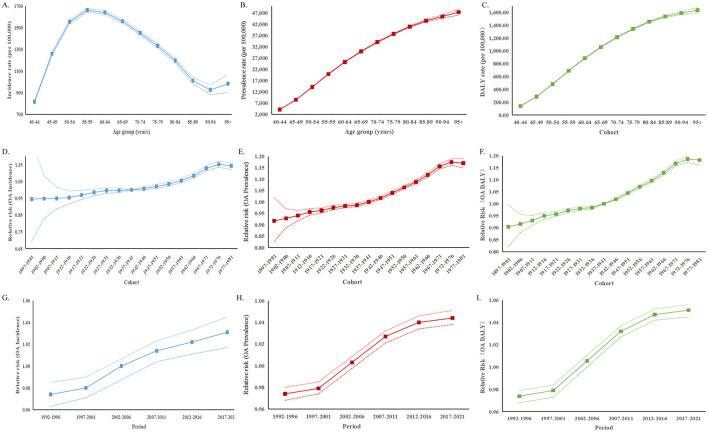
Age, period and cohort effects for OA incidence, prevalence and DALY among older people during 1992–2021. **(A–C)** Age effects are shown by the fitted longitudinal age curves of incidence, prevalence and DALY (per 100,000 person) adjusted for period deviations; **(D–F)** Period effects are shown by the relative risk of incidence, prevalence and DALY (relative ratio) and computed as the ratio of age-specific rates from 2002 to 2006 (the referent period); **(G–I)** Cohort effects are shown by the relative risk of mortality and computed as the ratio of age-specific rates from the 1897 to 1966 cohort, with the referent set at 1922–1926 cohort.

Figure 9 presents the APC model-derived estimates of the effects of age, period, and cohort on the burden of OA among older adults. The age effects are illustrated by longitudinal age curves that reflect the natural age-related trajectory of OA incidence, prevalence, and DALYs. Overall, the incidence was highest among the 55–60 years and generally declined with advancing age, with a notable increase observed in those aged over 90 years. In contrast, for prevalence and DALYs, the burden increased progressively with age, indicating greater accumulation of disease and disability in older age groups. The period effects represented by relative risks across different times, demonstrated a generally increasing trend for OA incidence, prevalence, and DALYs, suggesting a growing OA burden over time. The cohort effects, expressed as relative risks across successive birth cohorts, revealed that individuals born in more recent cohorts exhibit higher risks for OA incidence, prevalence, and DALYs. This indicates that younger generations are experiencing a progressively increasing burden of osteoarthritis.

**Figure 10:**
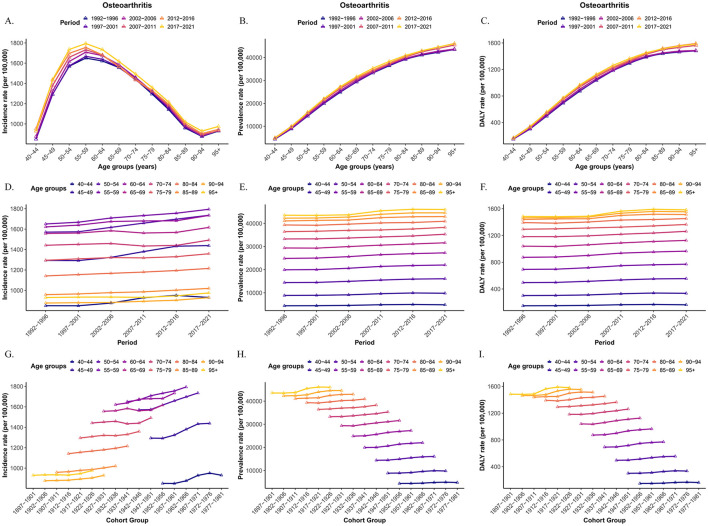
Trends of age-specific, period-based and cohort-based variation of OA incidence, prevalence and DALY rate among older people. **(A–C)** Age-specific OA incidence, prevalence and DALY rate; **(D–F)** period-based OA incidence, prevalence and DALY rate; **(G–I)** cohort-based OA incidence, prevalence and DALY rate.

Figure 10 presents a comprehensive illustration of the age-, period-, and cohort-specific trends in OA incidence, prevalence, and DALY rates among older adults. The age-related patterns of OA burden exhibited consistent trends across different periods. For any given age group, incidence, prevalence, and DALY rates were generally higher in more recent periods, reflecting a growing OA burden over time. The cohort-based analysis further revealed that within each age group, individuals born in more recent cohorts experienced a higher OA burden. Among individuals from the same birth cohort, OA incidence peaked in the 55–60 age group, whereas prevalence and DALY rates continued to increase with advancing age. Period-based variations showed that across all age groups, OA burden was greater in later periods, reinforcing the overall upward trend. Within each period, OA incidence peaked at ages 55–60, while prevalence and DALY rates steadily rose with age.

## Discussion

With the data derived from GBD 2021, we systematically analyzed trends in the incidence, prevalence, and DALYs of OA among people aged 55 years and older across 204 countries and territories from 1990 to 2021, with extended projections to 2050. To our knowledge, this is the first study to apply the APC modeling framework to assess time trends in the global burden of OA, with a specific focus on older populations. Our findings reveal several key insights that underscore the increasing challenge posed by OA worldwide.

OA continues to impose a substantial global disease burden among older adults, with an estimated 453.56 million aged 55 years and older living with OA in 2021, accounting for 30.39% of this population, and contributing to 16.05 million DALYs. Consistent increasing trends were observed in the incidence, prevalence, and DALYs of OA in aging populations during the past 3 decades. From 1990 to 2021, the EAPCs in ASIR, ASPR and ASDR suggest a progressive global expansion of the OA burden. These trends are likely driven by population aging, lifestyle changes, and increased life expectancy (17–19). Although high-SDI regions bore the greatest absolute burden, the rate of increase has attenuated in recent years. The decelerating trend in these regions may benefit from improved public health awareness, healthier lifestyles, and better access to healthcare services (20). In contrast, the most rapid increases were observed in middle-SDI regions, followed by low-middle SDI regions, possibly due to factors such as high levels of physical labor, limited diagnostic capacity, and inadequate awareness and implementation of preventive strategies (21, 22).

Sex disparities were consistently observed, with females consistently exhibiting higher ASDR than males across all SDI regions, particularly for knee and wrist OA. These differences may be explained by a combination of postmenopausal hormonal changes, sex-specific biomechanical or anatomical differences, and sociocultural roles. Declining estrogen levels in postmenopausal women have been linked to accelerated cartilage degeneration and increased OA severity (23, 24). Postmenopausal women experience a sharp decline in estrogen, which compromises its protective effect on joints, thereby accelerating degenerative changes. Anatomical and physiological differences, such as wider pelvis, larger Q-angle, lower bone mass, and reduced muscle strength, further increase the risk of both traumatic and non-traumatic OA in women (25–27). Additionally, women's disproportionate involvement in housework and caregiving may elevate their risk of joint injury and OA. Delayed healthcare-seeking behaviors among women may also contribute to later diagnosis and treatment, exacerbating the OA burden (28). Studies have also reported significantly lower limb muscle strength loss in women before age 70, which accelerates after age 60, potentially explaining the more severe and less reversible symptoms of knee OA in older females (29).

High BMI remains a critical modifiable risk factor for OA (30). In 2021, 35.8% of OA-related DALYs were attributable to high BMI; the attributable burden varied widely by region, reaching over 45.8% in high-income North America, but less than half that (20.9%) in South Asia. These differences likely reflect regional disparities in dietary habits, physical activity levels, and socioeconomic conditions. High-SDI countries commonly consume diets high in fat, salt, and ultra-processed foods, increasing the risk of obesity. In contrast, lower-SDI regions often experience food scarcity and higher levels of physical activity, reducing obesity-related OA risk (31). Several studies support that overweight, general obesity, and central obesity are key risk factors for OA in older adults. Central obesity, primarily characterized by increased abdominal and visceral fat, is associated with chronic low-grade inflammation and lipid accumulation, which contribute to cartilage degeneration and OA progression. Dyslipidemia, in turn, may impair blood supply to joint tissues, accelerating joint degradation and leading to higher OA prevalence among individuals with obesity and lipid disorders (32). Interestingly, adherence to the Mediterranean diet—common in Southern Europe, especially Italy—has been shown to reduce oxidative stress and lower OA risk. North American populations adhering to this dietary pattern also exhibited lower OA incidence (33–35).

From the perspective of the global burden of disease framework, the formation of the peak incidence rate of knee osteoarthritis among people aged 55–59 is the result of the combined effect of three forces: demographic transformation, changes in socio-economic models, and advancements in medical technology. Its impact transcends a single country or region and has become a significant challenge to global public health. The accelerated global aging process has directly expanded the base of susceptible people for knee osteoarthritis. The peak incidence rate among people aged 55–59, which is regarded as the “pre-aging stage”, reflects the differences in intergenerational health reserves. The group in early industrialized countries bears higher mechanical loads (such as manufacturing and agricultural labor) during their prime years, while the group in later developing countries is under the dual pressure of rising obesity rates and the popularization of sedentary lifestyles. This “differentiation in exposure history” leads to differences in peak patterns across different regions, but all point to the critical turning point of 55–59 years old.

The global labor market is transforming from physically intensive to knowledge-intensive. Although this has reduced occupational joint injuries, it has prolonged sedentary time, indirectly exacerbating muscle atrophy and joint load imbalance. Meanwhile, the delay in the retirement age keeps people aged 55–59 in a state of “high load - low repair”, accelerating the process of cartilage degeneration. The popularization of imaging techniques and the improvement of grassroots diagnosis and treatment standards have significantly increased the detection rate of early knee osteoarthritis, but they may also lead to the controversy of “overdiagnosis”. Some asymptomatic cartilage wear was included in the statistics, magnifying the observed value of the disease burden. Furthermore, the uneven distribution of global medical resources has further exacerbated regional disparities in the burden of diseases.

The APC model provided a comprehensive dissection of temporal trends in OA burden, revealing distinct contributions of age, period, and cohort effects to the observed epidemiological patterns. The greatest increases in OA burden occurred in the 55–59 age group, with subsequent attenuation but still positive trends in older age groups. Notably, individuals born in more recent cohorts exhibited higher relative risks of OA incidence, prevalence, and DALYs compared to earlier cohorts, potentially due to increased exposure to risk factors such as obesity, physical inactivity, or occupational strain. Physical activity is another effective strategy for preventing obesity and OA. Experts recommend a combination of aerobic and resistance exercises, personalized according to individual preferences, with the aim of alleviating pain, enhancing proprioception, and improving joint function (36). However, excessive physical activity may damage intra-articular soft tissues and increase OA risk (37). Period effects also indicate a temporal increase in OA burden, consistent with the global trend toward aging populations and improved diagnostic capabilities.

A particularly noteworthy finding of our study is the downward trend in ASPR and ASDR of OA among older adults in Denmark, contrary to the global upward trend. Literature suggests that this may be attributable to the implementation of the Good Life with Osteoarthritis in Denmark (GLA:D) program. GLA:D is an 8-week evidence-based treatment program for individuals with symptomatic knee or hip OA, consisting of patient education by clinicians and supervised exercise sessions led by physiotherapists. The program aims to improve confidence in physical activity, enhance self-efficacy, and increase awareness and motivation for managing OA symptoms (38, 39). It breaks through the traditional passive treatment model of “symptom control - end-stage surgery”, activates the joint compensation mechanism with exercise therapy, shifts the treatment focus to “function maintenance”, and enables patients to transform from disease receptors to self-managers, revealing that the key to reducing the burden of KOA lies in activating the patient's own repair potential. This plan, through behavioral intervention, helps patients establish the perception that “exercise is treatment”, significantly enhancing exercise compliance and achieving long-lasting effects. This suggests that the current global disease burden predictions may be overestimated. In addition, Denmark has incorporated it into the primary health care system, achieving wide coverage through policy incentives without the need for high-end equipment, providing a low-cost and replicable solution for middle - and low-income countries. The core lies in reconfiguring the allocation of medical resources through community integration to achieve a dual reduction in disease burden and medical costs.

Historically, pain and disability in older adults were often considered inevitable consequences of OA and aging. In the early stages of OA, when symptoms progress slowly, many patients simply endure the pain and passively accept the limitations imposed on their daily lives and work. Today, OA is increasingly recognized as a systemic condition that can be prevented and treated in its early stages. Like other chronic diseases, OA can benefit from comprehensive prevention and early intervention strategies. Modifiable risk factors such as joint injury, obesity, and impaired muscle function can be effectively addressed through primary and secondary prevention efforts (40).

There are various treatment methods for OA in middle-aged and older people. In terms of non-pharmaceutical treatments, physical therapies such as hot compresses, cold compresses, and electrical stimulation can relieve joint pain and stiffness. Exercise therapy, such as water sports and low-intensity aerobic exercises, can enhance muscle strength and improve joint range of motion. Weight loss is crucial for obese OA patients as it can reduce joint pressure. In terms of drug treatment, non-steroidal anti-inflammatory drugs can relieve pain quickly, but long-term use may cause adverse reactions in the gastrointestinal tract and other areas. Intra-articular injection of sodium hyaluronate can lubricate joints and nourish cartilage. Surgical treatment is mostly used for patients with severe conditions. Joint replacement surgery can effectively restore joint function, but the surgical risks and costs are relatively high. The incidence of OA is very high among the middle-aged and older population. If not handled properly, it will not only greatly affect an individual's daily life but also impose a heavy economic burden on the healthcare system. In this situation, accurately assessing the disease burden of OA among middle-aged and older people is of great significance. Clarifying the differences in burden among different regions and genders can help allocate medical resources rationally. Understanding the changing trends of disease burden can assess the effectiveness of existing treatment strategies, provide directions for optimizing treatment plans and developing new therapies, and ultimately improve the quality of life of middle-aged and older OA patients.

This study benefits from the extensive data coverage and standardized methodology of the GBD study, enabling robust comparisons across time and regions. However, the study has several limitations. First, the definitions of OA in GBD may vary across countries and periods. The identification of OA cases primarily captures diagnosed, severe cases and will miss milder or undiagnosed cases. Second, only high BMI was considered as a risk factor, while other important contributors such as joint injury, genetic predisposition, or occupational exposures were not included. Third, the accuracy of the estimates is contingent upon the quality and completeness of national health data, as well as the capacity of healthcare systems to diagnose and report OA, both of which vary widely across countries. In particular, data from low-income regions were often sparse or of limited quality, which may affect the reliability and precision of OA burden estimates at the global level.

This study also has some limitations. On the one hand, it needs to be further clarified and expanded. Studies rely heavily on radiology (Kelgren-Lawrence ≥2) and symptom criteria to define patients with osteoarthritis. Such a definition approach implies that the true prevalence of early-stage osteoarthritis and osteoarthritis with only pain manifestations and no significant imaging changes is likely to be underestimated, as some early-stage patients have not yet reached this imaging grading standard. Or if only pain symptoms were present without imaging examinations, and thus were not included in the research scope, this would have a certain impact on the representativeness of the research results for the overall osteoarthritis condition.

On the other hand, during the special period of the COVID-19 pandemic, many factors have hindered patients from seeking medical treatment at hospitals. Outpatient resources are relatively limited due to the demand for epidemic prevention and control, and many non-urgent medical examinations and diagnoses have been postponed. Moreover, out of fear of being infected with the novel coronavirus, patients also voluntarily avoid going to the hospital. These factors combined led to the fact that the number of diagnoses of osteoarthritis was likely underestimated within 1 or 2 years of the COVID-19 pandemic, and many potential patients failed to receive timely diagnosis. Future research can attempt to adopt more comprehensive diagnostic criteria, covering patients with early-stage and different manifestations of osteoarthritis, and take into account the influence of social and environmental factors in special periods on disease diagnosis, in order to further optimize the research and improve the scientific and accurate nature of the research results.

## Conclusions

This study utilized global epidemiological modeling to quantify the burden of osteoarthritis among middle-aged and older adults and provides valuable insights for developing tailored prevention strategies, health policies, and clinical management plans. Substantial disparities exist across countries, regions, and SDI levels. Recognizing and understanding these differences are crucial for designing targeted interventions that meet the unique needs of diverse populations. Through inclusive and region-specific public health strategies, the global health community can work toward mitigating the future burden of OA and improving the quality of life for affected individuals worldwide.
